# Single-base-resolution methylomes of *populus trichocarpa *reveal the association between DNA methylation and drought stress

**DOI:** 10.1186/1471-2156-15-S1-S9

**Published:** 2014-06-20

**Authors:** Dan Liang, Zhoujia Zhang, Honglong Wu, Chunyu Huang, Peng Shuai, Chu-Yu Ye, Sha Tang, Yunjie Wang, Ling Yang, Jun Wang, Weilun Yin, Xinli Xia

**Affiliations:** 1 College of Biological Sciences and Technology, National Engineering Laboratory for Tree Breeding, Beijing Forestry University, Beijing 100083, China; 2 BGI-Shenzhen, Building 11, Beishan Industrial Zone, Yantian District, Shenzhen, Guangdong, China; 3 BGI-Tianjin, E3 building, Airport Business Park, Tianjin Airport Economic Area,Tianjin, China

**Keywords:** Methylation, populus trichocarpa, drought tolerance, alternative splicing

## Abstract

**Background:**

DNA methylation is an important biological form of epigenetic modification, playing key roles in plant development and environmental responses.

**Results:**

In this study, we examined single-base resolution methylomes of *Populus *under control and drought stress conditions using high-throughput bisulfite sequencing for the first time. Our data showed methylation levels of methylated cytosines, upstream 2kp, downstream 2kb, and repeatitive sequences significantly increased after drought treatment in *Populus*. Interestingly, methylation in 100 bp upstream of the transcriptional start site (TSS) repressed gene expression, while methylations in 100-2000bp upstream of TSS and within the gene body were positively associated with gene expression. Integrated with the transcriptomic data, we found that all *cis*-splicing genes were non-methylated, suggesting that DNA methylation may not associate with *cis*-splicing. However, our results showed that 80% of *trans*-splicing genes were methylated. Moreover, we found 1156 transcription factors (TFs) with reduced methylation and expression levels and 690 TFs with increased methylation and expression levels after drought treatment. These TFs may play important roles in *Populus *drought stress responses through the changes of DNA methylation.

**Conclusions:**

These findings may provide valuable new insight into our understanding of the interaction between gene expression and methylation of drought responses in *Populus*.

## Background

*Populus *(*Populus *sp.) is an ideal model system for investigating molecular mechanisms of trees in response to environmental stresses, due to its advantages including rapid growth, high yield, easy propagation, importance in the economy and available genomic resources [1,2]. Drought is a major abiotic stress that limits the survival and growth of young poplar plants [3]. Therefore, many studies have focused on understanding drought responsive mechanisms in *Populus *[4-6].

DNA methylation is an important biological form of epigenetic modification. Currently, methylation analysis is widely used to explore various mechanisms underlying biological survival in plants [7,8], humans [9], and insects [10]. Plant genomes show extensive cytosine methylation at CG, CNG (N represents any nucleotide), and CHH (H represents A, C or T) sites [11]. Previous studies indicated that the effect of DNA methylation in plants refers to DNA methylation preventing DNA transcription by combining with the genomic sequence of transcription factors, and another refers to specific proteins (known as methyl-CpG binding proteins) binding with methylated DNA and acting as competitors of transcription factors. Complexes of proteins affect chromosome histone acetylation, leading to transcriptional inhibition [12,13]. Methylation induced by biotic stress is generally associated with the silencing of parasitic DNA and expression of resistant genes, while abiotic stress-induced methylation is supposed to be linked with the transcription factors which participate in numerous biochemical pathways involved in acclimatization and stress response in plants [14].

Numerous studies have shown that DNA methylation levels could be affected by plant stress in *Arabidopsis*, rice, pea and other plants [15-18], but few about trees. Uthup et al. have reported the identification of DNA methylation patterns and their putative relationship with abiotic stress in the tree crop *Hevea brasiliensis *[14]. The percentage of hypermethylated loci increased, and that of fully methylated loci clearly decreased in *Quercus ilex *trees exposed to drought [19]. However, no studies performed methylation analysis of *Populus *at the genomic level under drought stress by using high-throughput bisulfite sequencing. To improve our understanding of the resistance mechanisms at the molecular level in *Populus*, we investigated DNA methylomes of *Populus trichocarpa *under normal and drought conditions, focusing on epigenetic regulation of stress responses.

## Results

### Bisulfite sequencing of the *Populus trichocarpa *genome

To generate the single-base resolution methylome of *P. trichocarpa *under normal (i.e., well water; WW) and drought stress (i.e., water stress; WS) conditions, we applied the Illumina Hiseq 2000 platform for bisulfite sequencing of DNA extracted from leaves. Reads that aligned to the unmethylated lambda DNA, which were added to the total DNA before applying the bisulfite treatment, were used to calculate the conversion rate. The conversion rates of WW and WS were 99.40% and 99.45%, respectively. Two sets of raw data were obtained from sequencing, with the output of 16.86 giga base pair (Gb) in WW sample and 16.63 Gb in WS. We subsequently used a series of filter criteria to ensure the data quality, including trimming low-quality reads(reads that contain more than 50% unknown bases) and retaining unique-aligned reads. Finally, 72.96% of the reads in WW and 76.69% of the reads in WS were used for further analysis. These data were estimated to cover the whole genome with 28.45- and 29.93-fold sequencing depths (Additional file 1). To avoid the effects of individual SNPs, the online original reference (http://genome.jgi-psf.org/poplar/poplar.home.html) was modified by the resequence data.

### DNA methylation in *Populus trichocarpa*

**Figure 1:**
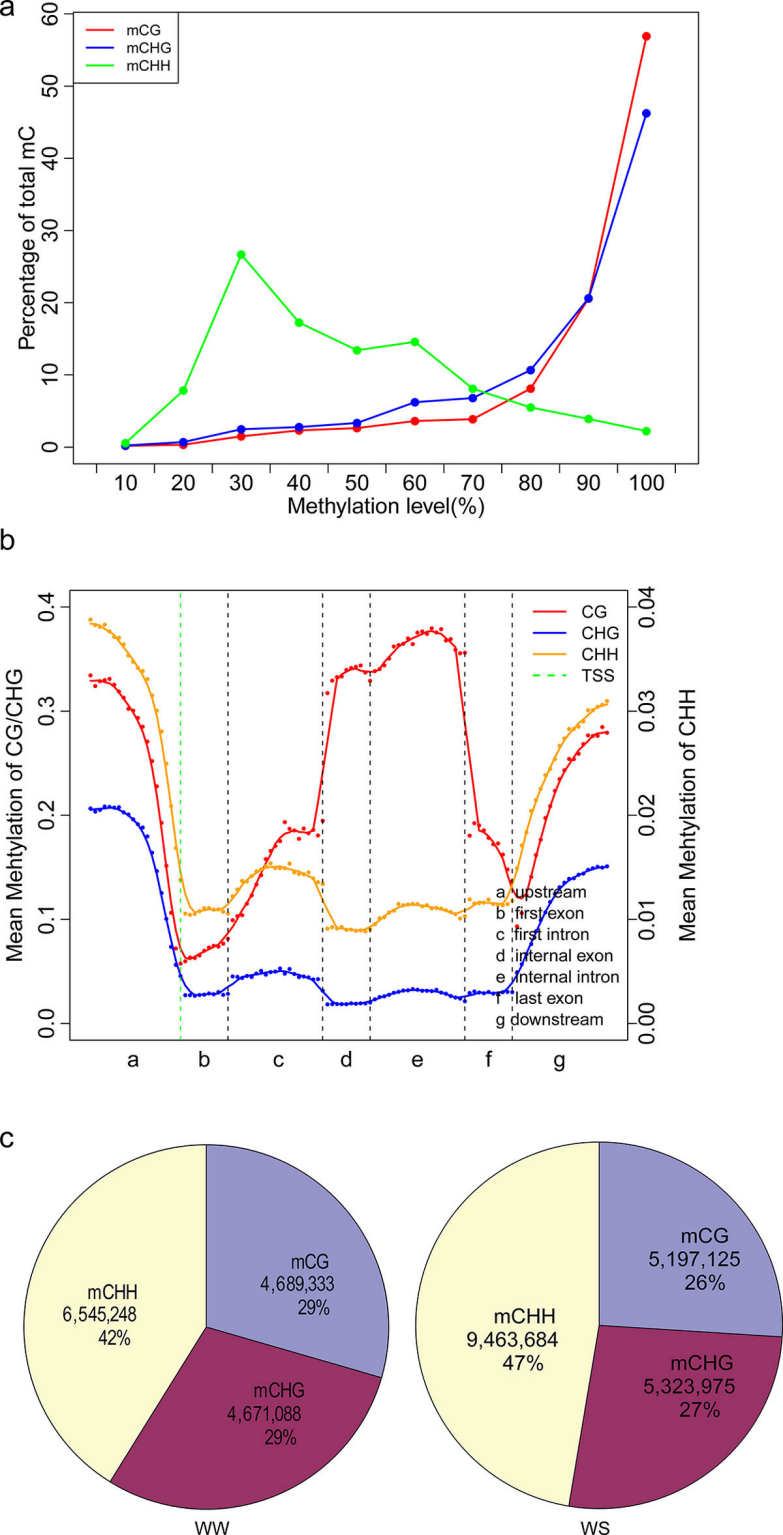
**The global pattern of *Populus *DNA methylomes**. (a) The percentage of methylated cytosines distribution in each sequence context (H=A, T, C). The *y *axis indicates the percentage methylated cytosines according to each methylation level which show on *x *axis.(methylation level = # of mC / # of C * 100%) (b) Distribution of CG, CHG and CHH methylation levels in each sequence context of gene related region, including upstream, first exon, first intron, internal exon, internal intron, last exon and downstream.. The *x *axis shows seven elements, the *y *axis represents average methylation levels of cytosines. The red dots represent average methylation levels of bins, the curve shows the average value of 5 bins with 1-bin step, and the green dotted line indicate TSSs (transcription start sites) (c) The percentage and absolute number of methylated cytosines that identified in WW(left) and WS(right) in each sequence context.

Our results showed that methylated cytosines (mCs) accounting for 10.04% of all cytosines in the whole genome under drought stress were significantly more than those (7.75 %) in WW (Additional file 2). Distribution of cytosine methylation levels showed that more proportions of mCG and mCHG have higher methylation levels as compared with mCHH (Figure 1-a). CG sites were much higher methylated in gene region than other sites (Figure 1-b). Additionally, our results showed that methylated sites were concentrated in the non-CG sites in the *Populus *genome, especially in mCHH (Figure 1-c), and the basic group of HH or mCHH tended to contain twofold more A or T (Additional file 3). Since the chromosome structure of the *P. trichocarpa *genome is imperfect, we concatenated the remaining fragments as the 20^th ^scaffold in addition to the existing 19 scaffolds and then analyzed the distribution of the mCs across chromosomes, which shows that there was a high methylated cytosine density in centromere regions that consisted of more repetitive sequences (Additional file 4).

**Figure 2:**
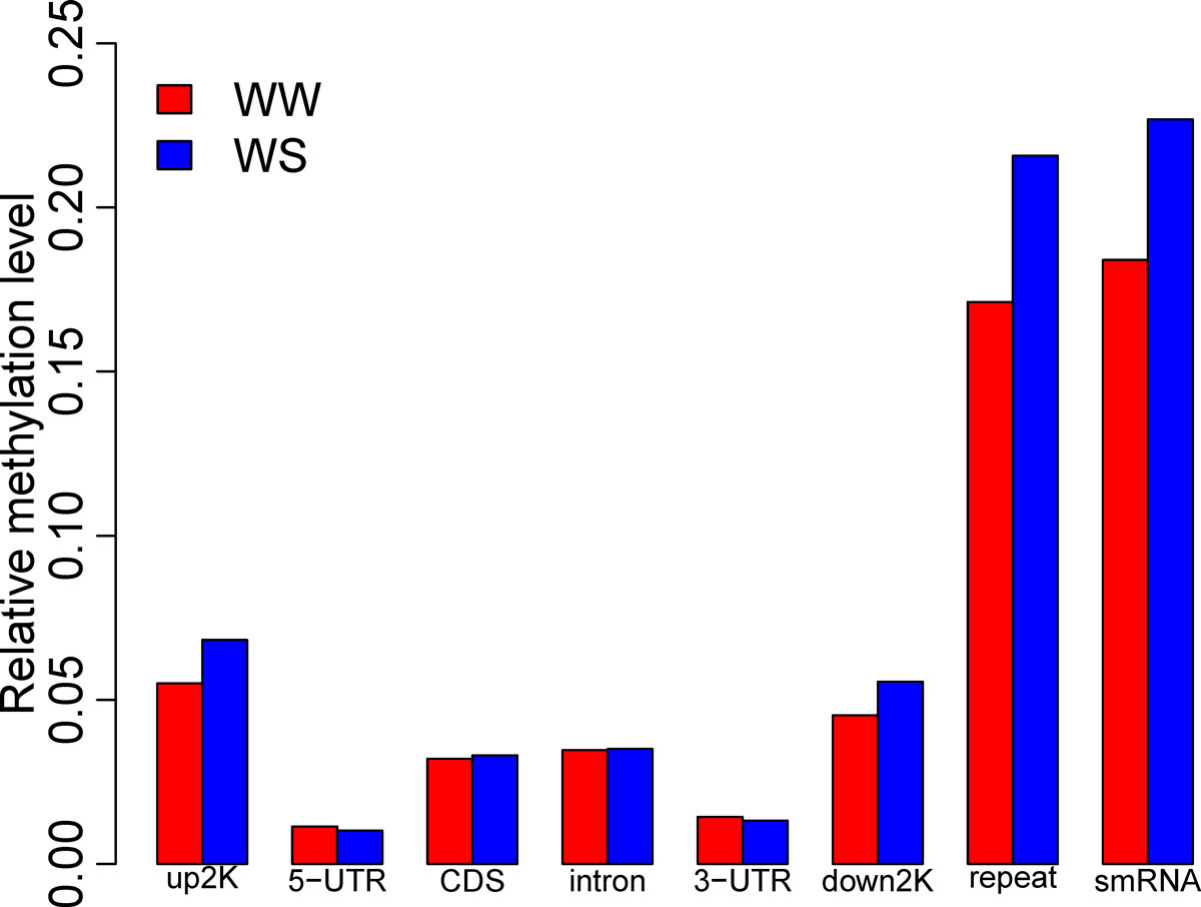
**Relative methylation levels of the corresponding genomic region**. The *y *axis shows the relative methylation level in each element of genomic region (*x *axis), which contains upstream, 5' UTR, CDS, Intron, 3' UTR, Downstream and repeative sequences.

The following analysis was to investigate the methylation profiles of coding sequences (CDSs), introns, untranslated regions (UTRs), small RNAs, repetitive DNAs under relative and *absolute *methylation standards [10]. We found that methylation levels in coding regions were higher than that in 5'- and 3'-UTRs. The methylation levels of repetitive regions were significantly higher than those of gene body regions (p < 7.71E-07, Wilcoxon RankSum test). Furthermore, the methylation levels *of *2000bp upstream of the transcriptional start site (*up2K)*, 2000bp downstream of the transcriptional termination site (down2K), and repetitive sequences were significantly higher after drought treatment (p < 0.001625, Wilcoxon RankSum test) *(*Figure. *2)*.

Gene Ontology analysis demonstrated biological processes related to biological regulation were enriched in both newly methylated and demethylated genes after drought stress as compared with the whole *Populus *genome, indicating that methylation or demethylation of these biological regulation related genes may play important roles in drought stress response of *Populus *(Additional file 5-a,b).

### Prediction and validation of splicing events in ***P. trichocarpa***

**Figure 3:**
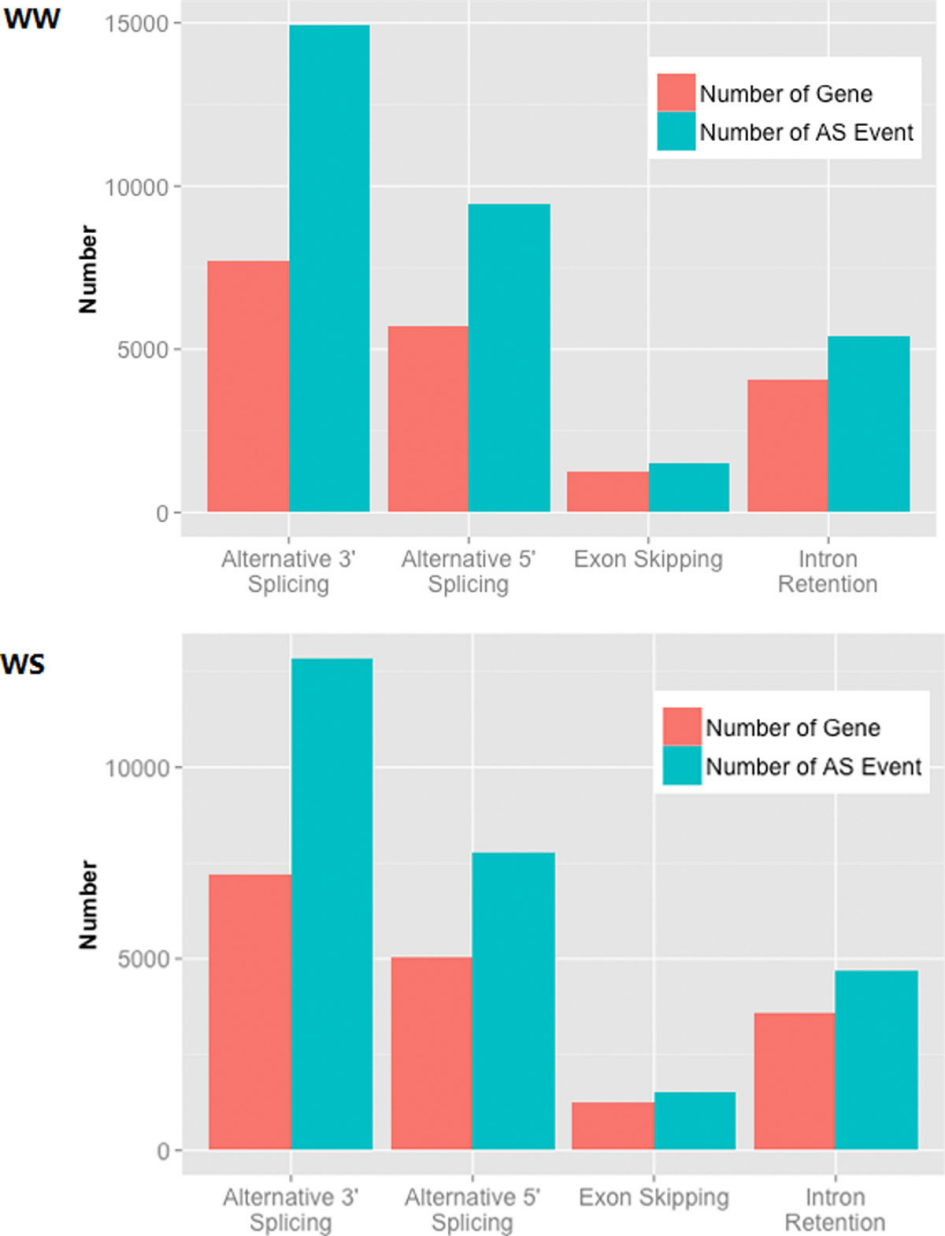
**The Histogram chart of alternative splicing events and genes identified in WW and WS**. The green columns indicate the number of alternative splicing events and the red columns show the total number of gene in each events.

We used transcriptome sequencing technology to further investigate the expression profiles of two samples (i.e., WW and WS). A total of 130,884,860 and 138,441,434 raw reads were generated, and 86,385,459 (66.00%) and 90,236,283 (65.18%) unique alignment reads were retained in WW and WS, respectively (Additional file 6). We analyzed two splicing forms of the genes, i.e., the *trans*-splicing and *cis*-splicing models. *Cis*-splicing occurs within a single transcript, which can produce multiple mRNA transcripts. *Trans*-splicing occurs at the bonding point, which is formed by two different genes [26]. Recently, alternative splicing and gene fusion were discovered by high-throughput sequencing in more species, such as human [27], rice [24] and *Arabidopsis*[28]. However, no information on alternative splicing in Populus was available. We found four alternative splicing types in *P. trichocarpa*: A) Exon skipping, B) intron retention, C) alternative 5 'splice sites, and D) alternative 3' splice sites, according to the classification of alternative splicing types in rice [24] and 11791 and 13251 alternative splicing genes in WW and WS, respectively. At least 20% of intron-containing genes in P. *trichocarpa *were spliced, which is lower than that (42%) in *Arabidopsis *[28]. More genes in WS were alternatively spliced in four alternative splicing types *(*Figure. *3)*, especially the alternative 3' splice sites were *more *increased, suggesting that alternative splicing can be regulated and associated with environmental stress as previous reports [29,30] and methylation may play more acting on alternative 3' splice sites after drought treatment in *Populus*. Particularly, in terms of the methylation level of 20 bp sequences containing alternative splicing sites, more than 80% of all alternative splicing sites in two samples were non-methylated, while none of the genes were methylated (Additional file 7-a). Interestingly, we found that 80% of the fusion genes were methylated and the splicing events were decreased after the drought treatment (Table 1). To verify the accuracy of the fusion gene, that identified 227 and 161 in WW and WS, we randomly selected 10 genes in the two groups of fusion materials to test by RT-PCR, six of which were verified (Additional file 7-b). The probability (6/10) was slightly lower than expected based on the accuracies in human and rice [24,27].

**Table 1:** Numbers of methylated and unmethylated gene fusions.

Sample	Methylated genes in the genome	Unmethylated genes in the genome	Fusion genes both methylated	Fusion genes both unmethylated	Fusion genes one methylated
WW	23983	8071	194	4	29
WS	22498	8895	130	11	20

### Effects of DNA methylation on gene expression in P. trichocarpa

To compare the effects of Populus DNA methylation on gene expression in two samples (i.e., WW and WS), we used transcriptome sequencing to determine the expression levels of methylated and unmethylated genes. Generally speaking, the expression levels of examined 17714 expressed genes were positively correlated with their methylation levels. We found that 7392 genes exhibited rising trends in methylation and expression levels after drought treatment. Methylation and expression levels were both reduced in 10322 genes under drought stress.

**Figure 4:**
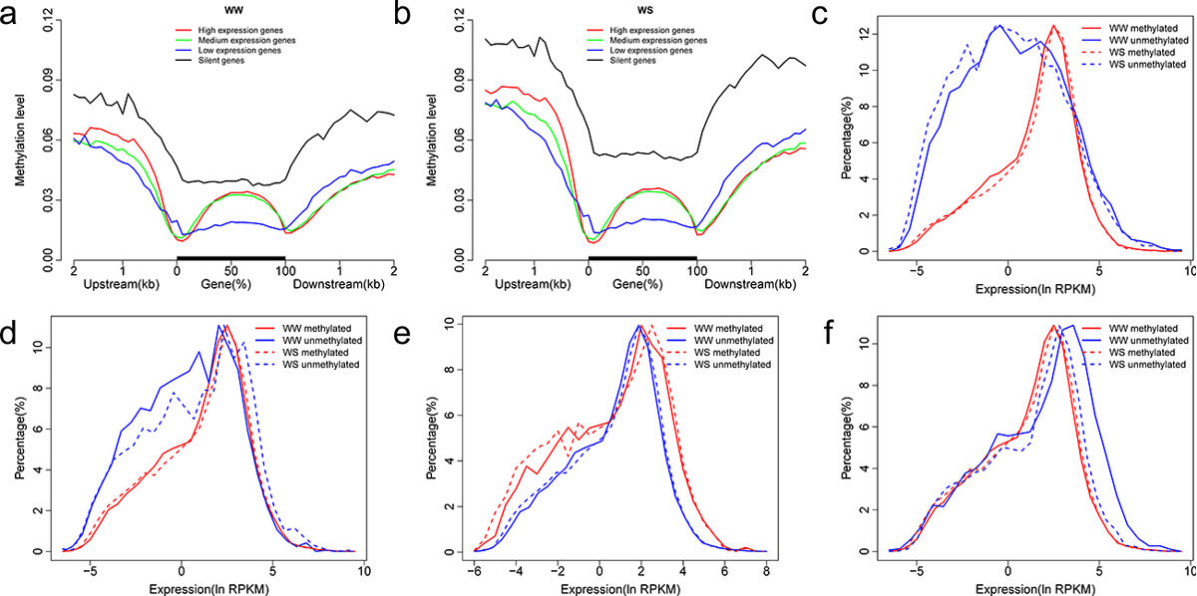
**The relationship between methylation level and gene expression**. (a) The methylation level trend (*y *axis) of four different gene clusters (High, medium, low, and silent genes, from the bottom one-third to the top one-third and non-expressed according to Transcriptome Sequencing data) in gene-associated region(*x *axis) in WW. (b) The methylation level trend (*y *axis) of four different gene clusters in gene-associated region(*x *axis) in WS (c) Distribution of methylated and unmethylated gene expression levels in gene-body. The horizontal axis represents expression level, and the vertical axis represents the percentage of genes on a particular expression level. (d) Distribution of methylated and unmethylated gene expression levels in upstream2k. (e) Distribution of methylated and unmethylated gene expression levels in 100bp upstream of the TSS. (f) Distribution of methylated and unmethylated gene expression levels in downstream2k.

To further analyze the relationship between gene expression and methylation levels, we divided these genes into four categories based on expression levels: High expression, medium expression, low expression, and silent genes, from the bottom one-third to the top one-third (Figure. 4-a). We found that silent genes had significantly higher methylation levels than expressed genes, indicating that gene silence may be caused by high methylation level (*p *= 6.8E-08, Wilcoxon Rank Sum test). For expressed genes, methylation levels in gene body and upstream region were positively correlated with gene expression (Additional file 8). Nonetheless, gene expression levels had negative correlations with methylation at transcription start sites (TSSs), transcriptional termination regions (TTRs) and downstream regions.

Gene-body methylation genes had significantly higher expression levels than body-unmethylated genes (*p *= 6.01E-07, Wilcoxon rank sum test) indicating a positive correlation between gene expression and gene-body methylation (Figure. 4-c). Interestingly, the results showed that upstream2k-methylated genes have significantly higher expression level than upstream 2k-unmethylated genes (*p *= 7.41E-05, Wilcoxon rank sum test) [31] (Figure. 4-d). However, further analysis was performed on the 100bp upstream of the transcriptional start site (TSS), and found that genes methylated in this region had significantly higher expression level than unmethylated genes (*p *= 8.6E-06, Wilcoxon rank sum test), indicating that methylation of this region induced gene expression (Figure.4-e). Coincident with the situation of 100 bp upstream of the TSS, downstream2k-unmethylated genes have higher expression level than downstream2k-methylated genes (Figure. 4-f).

As regards changes after drought treatment, the methylation levels of the silent genes increased significantly (*p *= 6.796E-08, Wilcoxon rank sum test) (Figure.4-a,b). The expression level of upstream2k-unmethylated genes was reduced, while it increased for genes methylated in 100bp upstream of TSS. Downstream2k-unmethylated genes have higher expression level after treatment. Gene-body methylated and unmethylated genes have no significant change.

### Differentially expressed and methylated transcription factors

Due to critical roles of transcription factors (TFs) in responses to external stimuli by influencing the expression of downstream targets, we identified differentially expressed and methylated TFs in *P. trichocarpa *according to two TF databases (i.e., DPTF (http://dptf.cbi.pku.edu.cn/index.php) [32] and PlnTFDB (http://plntfdb.bio.uni-potsdam.de/v3.0/) [33]. A total of 1156 TFs showed reduced methylation and expression levels after drought treatment. These TFs were distributed in 79 families, including MYB, AP2, WRKY, NAC, and bHLH. We also found that 690 TFs showed increased methylation and expression levels after drought treatment. Most of them belong to C3H, PHD, MYB, ARF, and bZIP families (Additional file 9).

Transposable elements (TEs) can influence gene regulation on a genomic scale by carrying potential transcription-regulating signals. To further analyze the mechanism underlying the TF response to drought stress by hypermethylation of transposons, we found 389 and 334 TFs were located by TEs in promoters and in gene bodies using the described method by Thornburg [34], respectively. The methylation levels of 138 TEs in promoters were elevated by drought treatment, while those of 251 TEs were reduced. The methylation levels of 163 TEs in gene body regions were elevated under drought treatment, while those of 171 TEs were reduced. These two kind of transcription factors were divided into 64 and 60 families (Additional file 10, 11), respectively, which were concentrated in both the major transcription factor families and some related to stress signal transduction, such as C2C2 and EIL proteins.

## Disscussion

Although the relationship between DNA methylation and gene expression has been explored in *Populus *and other plants [35,36], the resolution of genome-wide methylated cytosines requires more elaborate and comprehensive methylomic studies to characterize the functional effects of *Populus *DNA methylation. We obtained the single-base resolution methylomes of *Populus*, which was used to investigate the changes of DNA methylation under drought treatment via the high-throughput sequencing, and found the genome-wide methylation level in *P. trichocarpa *was slightly higher than that in *Arabidopsis *[24] but lower than humans [9]. Moreover, having found plausible patterns with our comprehensive dataset, our results have a number of implications, which will have a promising application in the future research on *Populus *and may give some cue on other plants' studies.

### The relationship of methylation and splicing events in ***P. trichocarpa***

The role of DNA methylation in alternative splicing is supported by Shukla et al. [37] in CD45 cells, as well as by Chodavarapu et al. in *Arabidopsis thaliana*, showing that DNA methylation is highly enriched in exons and may have an important role in alternative splicing [38]. We did not find any methylated sites in more than 80% of alternative splicing genes. In contrast with the previous results, DNA methylation is not enriched in exons and the methylation levels in exons were not significant higher than the other regions *in Populus*. Therefore, our results can't speculate that DNA methylation may play an important role in alternative splicing in *Populus *genome. By contrast, our results showed that all fusion genes were methylated, that inferred that this phenomenon might be associated with the mechanisms of two different splicing forms. Methylation may not affect the enzymatic reaction that results in alternative splicing, but can affect chromosomal rearrangements, RNA editing and other structural variations what might cause the occurrence of gene fusion.

Drought treatment has different effects on two different splicing forms, the number of genes with alternative splicing events was increased, while fusion genes were reduced after the drought treatment. Since the effects of drought on the complex network of signaling pathway *in Populus*, we can not point out the direct cause, but it can be speculated that it may be related to the formation or activity of cleavage enzyme, which main activates at 3 ' and 5 ' two positions.

### DNA methylation and gene expression

The way of the expression of genes affected by DNA methylation in plant is combining with specific protein, which competes with transcription factors. The complex causes the changes in chromosome histone acetylation, leading to the inhibition of transcription, that point was more prominent in poplar. In *Populus*, about 60% of silent genes were affected by methylation. After the drought treatment, the probability was increased, indicating that the influence of gene expression by methylation increased with the external environmental stimuli.

For expressed genes, methylation in 100 bp upstream of the TSS represses gene expression, which is consistent with the findings from *A. thaliana*[39], human [9] and rice [40], confirming that neighbouring upstream of TSS methylation is a general mechanism suppressing gene expression in eukaryotes. However, we found that methylation level in 100-2000 bp upstream was positively correlated with gene expression. It is interesting that the expression of genes methylated in 100bp upstream of TSS were affected after the drought treatment, which indicated that drought treatment reduced the chances of combining with specific protein and increased levels of gene expression. In the TTR (transcriptional termination regions) and the downstream region, methylation and expression were negatively correlated, indicating that they had significant effect on gene expression through interfering with transcriptional termination site.

Drought is one of the major environmental factors that affected gene expression by complex signaling networks, including cytosine methylation, histone acetylation and H3K9 methylation [17]. In *Populus*, the expression of gene was affected by drought on genetic elements in upstream and downstream. Combined with changes in methylation levels, 100 bp upstream of the TSS was focused. To further understand the relationship between methylation and drought, we need to make sure the genetic elements in different locations of upstream.

### Methylated transcription factors with TE in drought tolerance

Transcription factors involved in signal transduction related to various stresses, such as drought. Diverse biological activities were regulated directly or indirectly by these transcription factors. The demethylation of transcription factors under drought stress may reduce the stability of the gene, thereby affecting its expression. (For example, BZIP, WRKY, AP2 / EREBP and MYB, four major transcription factor families, play an important role in plant stress resistance, and many TFs of expression changed by methylation in our results also belong to them.).

The use of transposable elements (TEs) for reverse transcription, DNA cutting and ligation or DNA binding are well-documented [41]. Changes in transposition activity correlated with methylation were first described in maize [42,43]. Subsequently, in both plants and animals [44], the role of DNA methylation in transposons was directly tested by loss of DNA methylation (which is sufficient for the mobilization of transposons) [45]. TEs can influence gene regulation on a genomic scale by carrying potential transcription-regulating signals. When inserted in promoter regions, they can alter gene expression patterns by introducing new transcription factor binding sites [46]. Our data indicated that transcription factors that affect gene expression after drought treatment were affected by methylated transposons in *Populus*. We found methylated transposons in C2C2, WRKY MYB and other families that involved in signal transduction pathway of drought. When the environment changes, transposition frequency in plants increased. TEs were inserted into positive transcription factors and promoted expression, consequently increasing the expression of resistance genes. Insertion in some locations could inhibit the expression of positive transcription factors and decrease the resistance of a gene (such as by insertion of a negative transcription factor), which may have the opposite effect. Analysis of methylated transposons in transcription factors may increase our understanding of the specific mechanisms by which transcription factors regulate the stress response in plants.

## Conclusions

In this study, genome-wide DNA methylation sequencing of poplar leaves with WW and WS treatments were established using high-throughput technology. It confirmed that *cis*-splicing sites are unmethylated, while *trans*-splicing sites are methylated in poplar, to our knowledge this is the first report of an association between methylation and variable splicing. It further demonstrated that DNA methylation in the regulation of stress-responsive genes by identifying methylated transposable elements (TEs) in promoters and the gene body of transcription factors. Finally, the mechanism that the DNA methylation played on the gene expression, alternative splicing, and other phenomenon was still unclear based on current technology and experiment condition, otherwise our study pave the way for future discovering of methylation-associated mechanism in large-scale integrative multi-omics datasets.

## Methods

### Plant materials

*Populus trichocarpa *(Torr. & Gray) seedlings were planted separately into 10-L plastic pots filled with a mixture of clay, silt and sand (2:2:2, v/v). They were grown under well-watered (WW) conditions in a greenhouse at Beijing Forestry University for 2 months. The plants were supplemented with light for 15 h d^-1^, and temperature and humidity were kept constant. According to previous reports [20-22], the plantlets were divided into two groups, which were subjected to the following watering regimes: well-watered (WW) treatment (maintaining 100% of field capacity) and water-stress (WS) treatment (maintaining 25% of field capacity). Volumetric soil water content was kept at 24% in the WW treatment, while in the WS treatment the volumetric soil water content was kept at approximately 10%. Net photosynthetic rate, transpiration rate and leaf water potential (WP) were measured using the PsyPro WP data logger (Wescor) and Li-6400 Photosynthesis System (Li-Cor). After 5 weeks, plants of similar height (~75 cm) were selected for the experiment. Five replicates (four plantlets per replicate) were used to minimize random errors. Mature leaves from the same position (8-10th counting from the apex) of different individual plants were collected and frozen immediately in liquid nitrogen for DNA extraction.

### BS-Seq libraries construction and sequencing

Total DNA was extracted from the leaves collected from our experiment. Total DNA was prepared by proteinase K/phenol extraction following the manufacturer's instructions. The DNA was fragmented by sonication using a Bioruptor (Diagenode, Belgium) to a mean size of ~250 bp, followed by the blunt ending, dA addition to 3'-end, and adaptor addition following the manufacturer's protocol. The bisulfite conversion of the adaptor-added DNA was performed as described previously [23]. Ultra-high-throughput pair-end sequencing was performed using the Hiseq 2000 according to the manufacturer's instructions. Raw data were processed using Illumina base-calling pipeline.

### Mapping and processing of BS-Seq reads

Integrated with the *Populus *reference (v2.0) and resequencing data, we constructed a consensus sequence as a reference to align the methylation data. Because the strand-specific of methylation, two artificial genomes were construct, the T genome that whose cytosines had been converted to thymines and the A genome whose guanines had been converted to adenosines. After removing the low quality reads, all cytosines of reads1 were converted to thymines and all guanines of reads2 were converted to adenosines, and then all these reads were aligned to the T genome and A genome using SOAPaligner (v2.01) software with parameters -m 175 -x 325 -v(default 2), respectively. To increase the accuracy of methylation related analysis, reads that aligned to more than one positions were discarded.

All the unmethylated cytosine can be converted from cytosine to thymine during the process of bisulfite conversion, but the methylated cytosine will be the same. To remove the methylcytosines from the background noise which cause by the non-conversion of bisulfite, we use the conversion rate of lambda DNA input as a negative control was calculated following the formula: p = 1 - (# of methylated cytosines) / (# of cytosines). Using this value as a measure of the false mC discovery rate, mC was identified at each base position according to the binomial probability distribution, following the correction algorithm of Lister et al. [9].

### Transcriptome sequencing

For the synthesis of cDNA and Solexa sequencing, we prepared 45 μg of total RNA for treated and control sample at concentrations of approximately 1500 ng/μl. We then enriched the poly (A) mRNA using beads with Oligo (dT) and interrupted mRNA into short fragments with fragmentation buffer. Using these short fragments as templates, we synthesized first-strand cDNA with hexamer-primers and reverse transcriptase (Invitrogen). The second-strand cDNA was synthesized using buffer, dNTPs, RNaseH (Invitrogen) and DNA polymerase I (New England BioLabs). The short fragments were then purified using a QiaQuick PCR extraction kit and re- solved with EB buffer to finish the end reparation, and was connected using sequencing adaptors. After resolution by agarose gel electrophoresis, we selected fragments suitable for PCR amplification. We then constructed two paired-end libraries which were sequenced using an Illumina HiSeq 2000.

### Prediction of splicing events

The pair-end reads that aligned to two different genes were considered candidate fusion genes. To ensure the accuracy of detecting the fusion genes, PE reads that aligned to more than one location were discarded. Junction sequences were obtained by combining the exons of candidate genes. Junction reads and PE reads having only one end were compared to reference genes (5-bp overlap at least on the fusion point), and the candidate genes were supported by both PE reads and junction reads. The homologous candidate genes were detected and excluded, and the identification of alternative splicing sites was performed as described previously [24].

Collected potential splice sites by enumerating all possible pairs of donor sites (GT on the forward strand and AC on the reverse strand) and acceptor sites (AG on the forward strand and CT on the reverse strand) inside intron regions. Secondly, filtered all potential splice sites through the information of supported reads. At least two unique mapped reads convered on the junction site and having a minimum of five bases on both sides of the junction. Finally, categorized all these splice sites into seven types including exon skipping (ES), intron retention (IR), alternative 5' splice site (A5SS), alternative 3' splice site (A3SS), mutually exclusive exons (MXE), alternative first exons (AFE), and alternative last exons (ALE).

### Gene ontology (GO) analysis

GO annotations of poplar genes were downloaded from (http://bioinfo.cau.edu.cn/agriGO/download/item2term_46). GO comparative analyses between interested gene groups were performed using BGI WEGO (http://wego.genomics.org.cn/cgi-bin/wego/index.pl). GO enrichment analysis was performed using Blast2GO and agriGO (http://bioinfo.cau.edu.cn/agriGO/) with Fisher's exact test [25].

## Competing interests

The authors declare that they have no competing interests.

## Authors' contributions

DL PS designed and conducted the experiments, DL HL CH PS ZZ ST analyzed the data, DL HLPS WY XX drafted the manuscript, WY XX supervised the project. All authors have read and approved the final version of this manuscript.

## Supplementary Material

Additional file 1 Description of the data for the *Populus *of two treatments Click here for file

Additional file 2 Three methylation patterns of *Populus* Click here for file

Additional file 3 **Seqlogo of the sequences proximal to DNA methylation cytosines**. One stack for each position in these two sequence contexts (CHG, CHH), The overall height of the stack indicates the sequence conservation at that position, while the height of bases within the stack indicates the relative frequency of each base at that position. (red = T, green = A, blue = C, yellow = G) Click here for file

Additional file 4 Density of methylcytosines identified in each chromosome in *Populus*. Red dots indicate the density of all methylcytosines in 10 kb windows. The top panel shows the Watson strand information and the bottom panel displays crick strand information. Click here for file

Additional file 5 **GO enrichment analysis newly methylated and demethylated genes**. Click here for file

Additional file 6 Description of the data for the *Populus *of two treatments by Transcriptome Sequencing Click here for file

Additional file 7 **Results of two splicing forms**. (a) Four kinds of alternative splicing types were compared to each other on methylation level; (b) the PCR result of the fusion genes verification. Click here for file

Additional file 8 **The correlation between gene methylation and gene expression**. The upstream, gene body and downstream were split into 20 bins that lay on x-axis for investigating the spearman rank correlation(y-axis) between methylation and expression. Red line stands for WW and blue line stands for WS. Click here for file

Additional file 9 Category of co-regulated transcription factors Click here for file

Additional file 10 Category of transcription factors with methylated TE that located in promoter Click here for file

Additional file 11 Category of transcription factors with methylated TE that located in gene Click here for file

## Declarations

Publication charges for this work were supported by the National Natural Science Foundation of China (31070597, 31270656), the fund from the Ministry of Science and Technology of China (2009CB119101), and the Scientific Research and Graduate Training Joint Programs from BMEC (Stress Tolerance and DNA Methylation in Populus).

This article has been published as part of *BMC Genetics *Volume 15 Supplement 1, 2014: Selected articles from the International Symposium on Quantitative Genetics and Genomics of Woody Plants. The full contents of the supplement are available online at http://www.biomedcentral.com/bmcgenet/supplements/15/S1.
