# Supplementary material for: Single-base-resolution methylomes of populus trichocarpa reveal the association between DNA methylation and drought stress
Source: BMC Genet. 2014 Jun 20;15(Suppl 1):S9. doi: 10.1186/1471-2156-15-S1-S9 (PMC4118614; doi:10.1186/1471-2156-15-S1-S9)
Supplement: Additional file 1 — Description of the data for the Populus of two treatments [file 1471-2156-15-S1-S9-S1.docx]

Additional file 1 Description of the data for the *Populus* of two treatments

| Sample | WW | WS |
| --- | --- | --- |
| Raw reads (M) | 228.35 | 225.83 |
| data production (Gb) | 16.86 | 16.63 |
| Effective reads (M) | 166.61 | 173.18 |
| Effective production (Gb) | 11.90 | 12.53 |
| Average genome coverage | 28.45 | 29.93 |
