# Supplementary material for: Single-base-resolution methylomes of populus trichocarpa reveal the association between DNA methylation and drought stress
Source: BMC Genet. 2014 Jun 20;15(Suppl 1):S9. doi: 10.1186/1471-2156-15-S1-S9 (PMC4118614; doi:10.1186/1471-2156-15-S1-S9)
Supplement: Additional file 2 — Three methylation patterns of Populus [file 1471-2156-15-S1-S9-S2.docx]

Additional file 2 Three methylation patterns of *Populus*

| Sample | C | CG | CHG | CHH |
| --- | --- | --- | --- | --- |
| WW | 7.75 | 28.9 | 15.41 | 3.55 |
| WS | 10.04 | 35.16 | 20.14 | 4.82 |
