# Supplementary material for: Single-base-resolution methylomes of populus trichocarpa reveal the association between DNA methylation and drought stress
Source: BMC Genet. 2014 Jun 20;15(Suppl 1):S9. doi: 10.1186/1471-2156-15-S1-S9 (PMC4118614; doi:10.1186/1471-2156-15-S1-S9)
Supplement: Additional file 3 — Seqlogo of the sequences proximal to DNA methylation cytosines. One stack for each position in these two sequence contexts (CHG, CHH), The overall height of the stack indicates the sequence conservation at that position, while the height of bases within the stack indicates the relative frequency of each base at that position. (red = T, green = A, blue = C, yellow = G) [file 1471-2156-15-S1-S9-S3.docx]

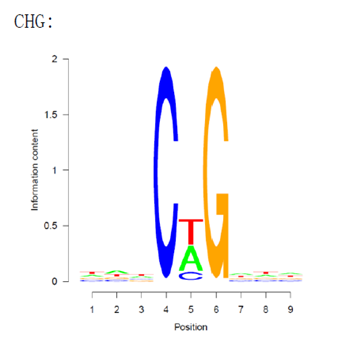

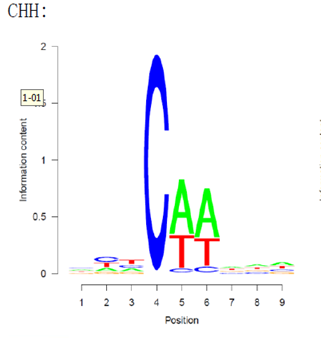


Additional file 3 Seqlogo of the sequences proximal to DNA methylation cytosines. one stack for each position in these two sequence contexts (CHG, CHH), The overall height of the stack indicates the sequence conservation at that position, while the height of bases within the stack indicates the relative frequency of each base at that position. (red = T, green = A, blue = C, yellow = G)
