# Supplementary material for: Single-base-resolution methylomes of populus trichocarpa reveal the association between DNA methylation and drought stress
Source: BMC Genet. 2014 Jun 20;15(Suppl 1):S9. doi: 10.1186/1471-2156-15-S1-S9 (PMC4118614; doi:10.1186/1471-2156-15-S1-S9)
Supplement: Additional file 4 — Density of methylcytosines identified in each chromosome in Populus. Red dots indicate the density of all methylcytosines in 10 kb windows. The top panel shows the Watson strand information and the bottom panel displays crick strand information. [file 1471-2156-15-S1-S9-S4.docx]

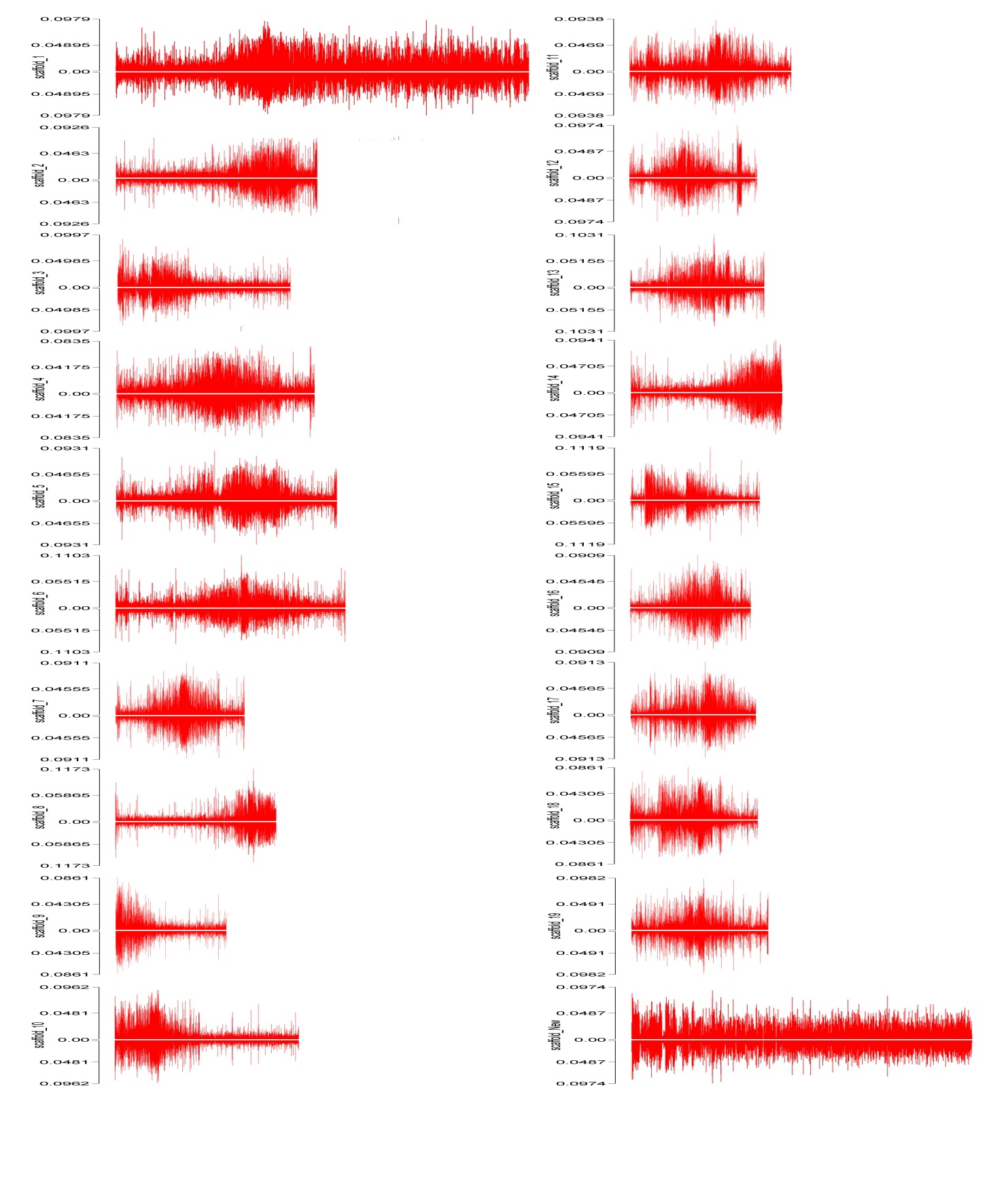


Additional file 4 Density of methylcytosines identified in each chromosome in *Populus*. Red dots indicate the density of all methylcytosines in 10 kb windows. The top panel shows the Watson strand information and the bottom panel displays crick strand information.
