# Supplementary material for: Single-base-resolution methylomes of populus trichocarpa reveal the association between DNA methylation and drought stress
Source: BMC Genet. 2014 Jun 20;15(Suppl 1):S9. doi: 10.1186/1471-2156-15-S1-S9 (PMC4118614; doi:10.1186/1471-2156-15-S1-S9)
Supplement: Additional file 6 — Description of the data for the Populus of two treatments by Transcriptome Sequencing [file 1471-2156-15-S1-S9-S6.docx]

Additional file 6 Description of the data for the poplar of two treatments by Transcriptome Sequencing

| Map to Genome | WW (reads number) | percentage | WS (reads number) | percentage |
| --- | --- | --- | --- | --- |
| Total Reads | 130884860 | 100.00% | 138441434 | 100.00% |
| Total Mapped Reads | 89690336 | 68.53% | 93478843 | 67.52% |
| unique match | 86385459 | 66.00% | 90236283 | 65.18% |
| multi-position match | 3304877 | 2.53% | 3242560 | 2.34% |
| Total Unmapped Reads | 41194524 | 31.47% | 44962591 | 32.48% |
