# Supplementary material for: Single-base-resolution methylomes of populus trichocarpa reveal the association between DNA methylation and drought stress
Source: BMC Genet. 2014 Jun 20;15(Suppl 1):S9. doi: 10.1186/1471-2156-15-S1-S9 (PMC4118614; doi:10.1186/1471-2156-15-S1-S9)
Supplement: Additional file 7 — Results of two splicing forms. (a) Four kinds of alternative splicing types were compared to each other on methylation level; (b) the PCR result of the fusion genes verification. [file 1471-2156-15-S1-S9-S7.docx]

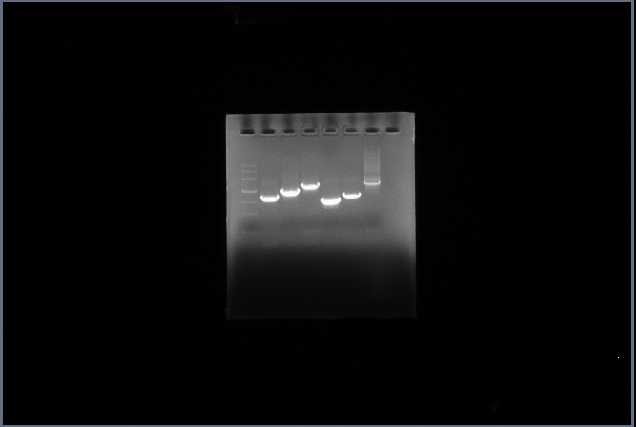


Additional file 7 Results of two splicing forms. (a) Four kinds of alternative splicing types were compared to each other on methylation level; (b) the PCR result of the fusion genes verification.

**b**

1500bp

**a**
