# Supplementary material for: Single-base-resolution methylomes of populus trichocarpa reveal the association between DNA methylation and drought stress
Source: BMC Genet. 2014 Jun 20;15(Suppl 1):S9. doi: 10.1186/1471-2156-15-S1-S9 (PMC4118614; doi:10.1186/1471-2156-15-S1-S9)
Supplement: Additional file 8 — The correlation between gene methylation and gene expression. The upstream, gene body and downstream were split into 20 bins that lay on x-axis for investigating the spearman rank correlation(y-axis) between methylation and expression. Red line stands for WW and blue line stands for WS. [file 1471-2156-15-S1-S9-S8.docx]

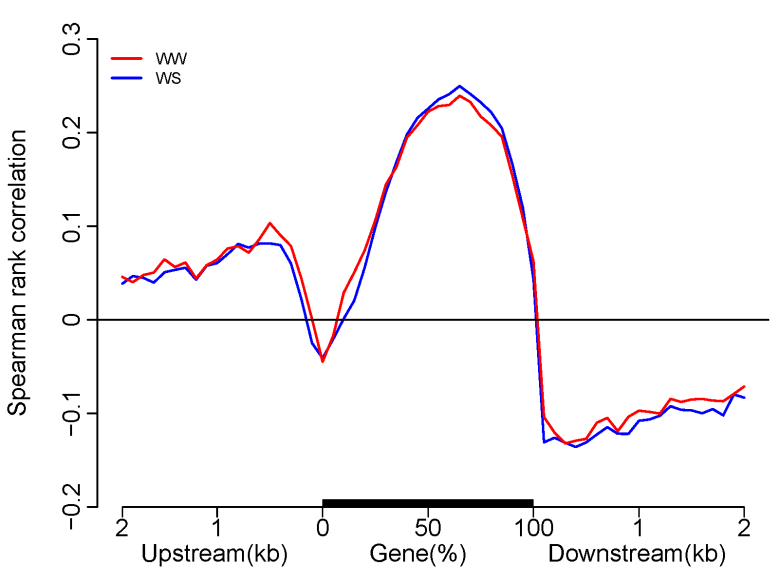


Additional file 8 The correlation between gene methylation and gene expression. The upstream, gene body and downstream were split into 20 bins that lay on x-axis for investigating the spearman rank correlation（y-axis）between methylation and expression. Red line stands for WW and blue line stands for WS.
